# The Isolation, Structural Characterization and Anti-Inflammatory Potentials of Neutral Polysaccharides from the Roots of *Isatis indigotica* Fort.

**DOI:** 10.3390/molecules29112683

**Published:** 2024-06-05

**Authors:** Yu Shen, Shihao Wu, Mingming Song, Huiming Zhang, Hong Zhao, Lili Wu, Hongbo Zhao, Hongbin Qiu, Yu Zhang

**Affiliations:** 1 Heilongjiang Provincial Key Laboratory of New Drug Development and Pharmacotoxicological Evaluation, College of Pharmacy, Jiamusi University, Jiamusi 154007, China; shenyu@jmsu.edu.cn (Y.S.); 228153048@stu.jmsu.edu.cn (S.W.); 15765339879@163.com (M.S.); banana_5016@163.com (H.Z.); zhaohong1981@jmsu.edu.cn (H.Z.); h42003@163.com (L.W.); 2 College of Rehabilitation Medicine, Jiamusi University, Jiamusi 154007, China; zhaohongbo@jmsu.edu.cn

**Keywords:** *Isatis indigotica* Fort., neutral polysaccharides, structural characterization, anti-inflammatory

## Abstract

Polysaccharides have been assessed as a potential natural active component in Chinese herbal medicine with anti-inflammatory properties. However, the complex and indefinite structures of polysaccharides limit their applications. This study explains the structures and anti-inflammatory potentials of three neutral polysaccharides, RIP-A1 (M_w_ 1.8 × 10^4^ Da), RIP-B1 (M_w_ 7.4 × 10^4^ Da) and RIP-B2 (M_w_ 9.3 × 10^4^ Da), which were isolated from the roots of *Isatis indigotica* Fort. with sequenced ultrafiltration membrane columns, DEAE-52 and Sephadex G-100. The planar structures and microstructures of RIP-A1, RIP-B1 and RIP-B2 were further determined by HPGPC, GC–MS, methylation analysis, FT-IR, SEM and AFM, in which the structure of RIP-A1 was elucidated in detail using 1D/2D NMR. The Raw 264.7 cells were used for the anti-inflammatory activity in vitro. The results showed that RIP-A1, RIP-B1 and RIP-B2 are all neutral polysaccharides, with RIP-A1 having the smallest M_w_ and the simplest monosaccharide composition of the three. RIP-A1 is mainly composed of Ara and Gal, except for a small quantity of Rha. Its main structure is covered with glycosidic linkages of T-*α*-Ara*f*, 1,2-*α*-Rha*p*, 1,5-*α*-Ara*f*, T-*β*-Gal*p*, 1,2,4-*α*-Rha*p*, 1,3,5-*α*-Ara*f* and 1,6-*β*-Gal*p* with 0.33:0.12:1.02:0.09:0.45:11.41:10.23. RIP-A1 significantly inhibited pro-inflammatory cytokines (NO, TNF-α, IL-6 and IL-1β) and increased anti-inflammatory cytokines (IL-4) in LPS-stimulated RAW 264.7 cells. Moreover, RIP-A1 could significantly inhibit the mRNA expression of TNF-α, IL-6 and L-1β. It could also activate IKK, p65 and IκBα (the components of the NF-κB signaling pathway). In conclusion, the above results show the structural characterization and anti-inflammatory potentials of RIP-A1 as an effective natural anti-inflammatory drug.

## 1. Introduction

Inflammation is an intrinsic immune response triggered by infection or tissue injury [1]. The local symptoms of inflammation include redness, swelling, heat, pain and loss of function, sometimes accompanied by fever. Increasing evidence indicates a strong correlation between excessive inflammation and several disorders, such as pulmonitis, carditis, gastritis, arthritis, enteritis and cancer [2,3,4,5]. Inflammatory stimuli such as lipopolysaccharide (LPS) could prompt macrophages to secrete pro-inflammatory mediators and cytokines, which cause tissue responses in different phases of inflammation [6]. Toll-like receptor 4 (TLR4) is activated and transmits signals through LPS, resulting in the activation of nuclear factor κappaβ (NF-κB) and its subsequent translocation into the nucleus [7]. After that, NF-κB can bind to the promoter regions of genes that produce inflammatory mediators. The NF-κB signaling system plays a crucial role in inflammation and innate immune responses, serving as a critical anti-inflammatory pathway [8]. Research into efficacious natural anti-inflammatory drugs has garnered significant attention in recent years as a novel approach to some diseases.

Polysaccharides are a group of beneficial macromolecules of natural products that have been reported to perform various activities, like anti-oxidant, anti-inflammatory, anti-tumor, anti-microbial and immunomodulatory activities [9,10,11,12]. Fortunately, polysaccharides are widely distributed in plants, animals, microorganisms and fungi. Recently, it has been shown that polysaccharides have abundant resources, low toxicity and significant activity, which have made them an appealing subject for investigation [13,14,15,16]. Understanding the structures of polysaccharides is crucial for investigating their uses with more effectiveness. Although the structure–activity relationship of polysaccharides is not clear, molecular weight (M_w_), monosaccharide composition, glycosidic linkages and configuration are believed to influence the activity of polysaccharides [17,18]. Some research utilizes in vitro investigations using RAW 264.7 cells to ascertain the anti-inflammatory effects of polysaccharides [19,20,21]. Research has indicated that polysaccharides with low molecular weights and small numbers of monosaccharide types can inhibit the synthesis of pro-inflammatory mediators when exposed to LPS, leading to an anti-inflammatory effect. Strong anti-inflammatory effects and wound healing are provided by the hydrolyzed low-molecular weight polysaccharide from *Enteromorpha prolifera* (LPEP). The anti-inflammatory effectiveness of the *Lycium barbarum* polysaccharide (LBP) fraction is significantly improved by its high levels of arabinose and galactose. Moreover, the anti-inflammatory effects of polysaccharides differ depending on their specific glycosidic bond configurations or three-dimensional structures. The anti-inflammatory effects of fungal polysaccharides are primarily due to their glycosidic linkages. The differences in the molecular weight distributions and three-dimensional structures of crude polysaccharides (CP) and water-soluble nondigestible polysaccharides (NDP) may contribute to their anti-inflammatory properties [22,23,24,25,26].

Isatidis Radix is the dried root of *Isatis indigotica* Fort. (also known in Chinese as Ban-Lan-gen), which is mainly produced in northern China, India and Southeast Asia. Traditionally, it is believed that Isatidis Radix has been applied in clearing heat and detoxifying for centuries [27]. It is used in clinical practice for the therapy of influenza, sore throats, inflammation and viral pneumonia [28,29,30,31]. Isatidis Radix has a diverse range of beneficial compounds, including alkaloids, organic acids, flavonoids, polysaccharides and glycosides [32,33,34]. In the last few decades, alkaloids have become generally recognized as one of the main active constituents of this herb [35]. Furthermore, the advancement of polysaccharide pharmacology and structural characterization has revealed promising therapeutic advantages in the field of medicine [36]. Isatidis Radix polysaccharides (RIP) are macromolecules that exhibit a range of biological activities, including combating the hepatitis B virus [37], improving obesity and acting as anti-oxidants and anti-inflammatory agents [38,39]. Despite the acquisition of various RIPs in recent years, the structural characteristics of neutral polysaccharides, their anti-inflammatory efficacy and the association between these traits remain unknown [40,41]. Thus, it was postulated that the neutral polysaccharide obtained and refined from the roots of *Isatis indigotica* Fort. may possess anti-inflammatory properties due to its polysaccharide structure.

This work employed a range of separation techniques and structural identification methods to isolate and structurally characterize neutral polysaccharides from the roots of *Isatis indigotica* Fort. Various analyses were carried out to investigate the structural properties of these active polysaccharides, offering empirical support for the future advancement and utilization of *Isatis indigotica* Fort. Moreover, this work investigated the structure–function relationships of polysaccharides by examining their anti-inflammatory characteristics, which is crucial for assessing the potential of these molecules for novel anti-inflammatory drugs. In summary, this research provides a thorough comprehension of RIP, hence facilitating their practical implementation in the medical sector.

## 2. Results and Discussion

### 2.1. Extraction, Isolation and Purification of Polysaccharide

**Figure 1:**
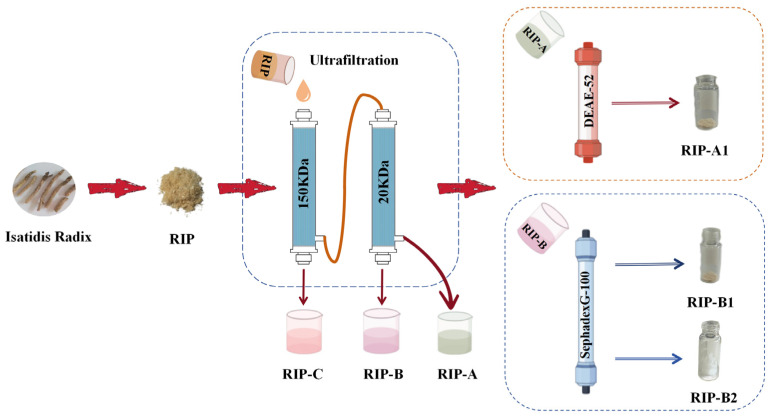
Workflow of isolation and purification from the roots of *Isatis indigotica* Fort.

Polysaccharides from the roots of *Isatis indigotica* Fort. were isolated and purified, as schematically depicted in Figure 1. The crude polysaccharide RIP (3.92%, wt%) was obtained from *Isatis indigotica* Fort. via a series of processes including deracination, hot water extraction, ethanol precipitation and freeze-drying. Then, the RIP was sequentially graded by 150 kDa and 20 kDa ultrafiltration membranes to collect three sub-fractions, RIP-A (54.32%, wt%; <20 kDa), RIP-B (35.70%, wt%; 20–150 kDa) and RIP-C (9.98%, wt%; >150 kDa). The sugar contents of RIP-A–C were 67.04%, 78.49% and 59.32%, respectively. Among the three fractions, it was found that RIP-A had the largest proportion, while RIP-B had the highest sugar content. Therefore, RIP-A and RIP-B were primarily considered for isolation and purification. As shown in Figure S1A, RIP-A was purified by the DEAE-52 column to get five sub-fractions (RIP-A1–5). RIP-B was chromatographed on the Sephadex G-100 column (GE Healthcare Life Science, Uppsala, Sweden) to obtain two sub-fractions named RIP-B1 and -B2 (Figure S1B). Moreover, three homogeneous polysaccharides—RIP-A1 (33.1%, wt%), RIP-B1 (33.3%, wt%) and RIP-B2 (19.6%, wt%)—were acquired, which were all observed as a single and symmetrical peak by a high-performance gel permeation chromatography (HPGPC) chromatogram (Figure S2A–C).

### 2.2. Molecular Weight and Monosaccharide Composition

**Figure 2:**
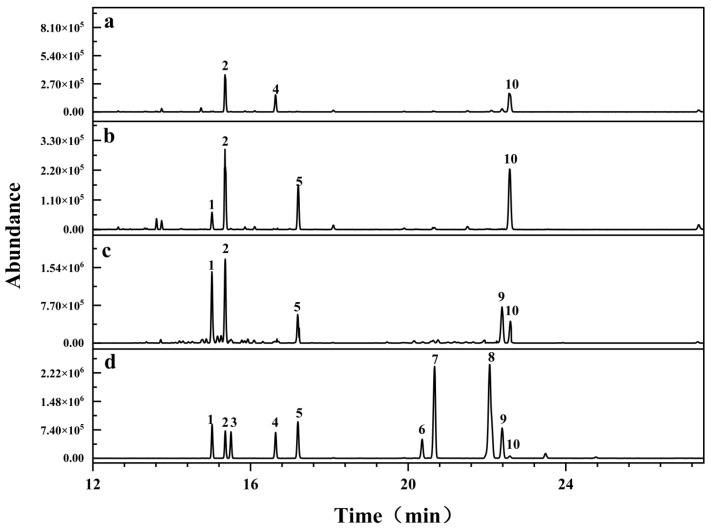
Monosaccharide compositions of RIP-A1, RIP-B1 and RIP-B2. GC–MS diagram of RIP-A1, RIP-B1 and RIP-B2 monosaccharide compositions. (**a**): RIP-A1, (**b**): RIP-B1, (**c**): RIP-B2, (**d**): mixed monosaccharide standards, 1, Xyl; 2, Ara; 3, Rib; 4, Rha; 5, Fuc; 6, GlcUA; 7, GalUA; 8, Man; 9, Glc; 10, Gal.

RIP-A1, RIP-B1 and RIP-B2 exhibited elution times of around 15.583, 12.535 and 12.038 min, respectively, in the HPGPC chromatogram. According to the calibration curve of standard dextran (log M_w_ = −0.2027x + 7.4201; R^2^ = 0.9908), the average molecule weights of RIP-A1, RIP-B1 and RIP-B2 were calculated as 1.8 × 10^4^ Da, 7.4 × 10^4^ Da and 9.3 × 10^4^ Da, respectively. Low M_w_ polysaccharides were found to have higher physiological activity among a variety of polysaccharide components. Polysaccharides with a lower M_w_ exhibited a higher tendency to traverse cell membranes and induce biological responses [42,43]. Hence, RIP-A1 might potentially be the most active one among them. In this study, the monosaccharide compositions of RIP-A1, RIP-B1 and RIP-B2 were examined via gas chromatography–mass spectrometry (GC–MS)-based trimethylsilyl-alditol (TMSA) derivatives [44]. As shown in Figure 2, it was found that RIP-A1 consisted of Ara, Rha and Gal in a molar proportion of 2.19:1.00:1.70. RIP-B1 consisted of Xyl, Ara, Fuc and Gal in a molar proportion of 1.00:5.00:2.83:5.58. RIP-B2 consisted of Xyl, Ara, Rib, Fuc, Glc and Gal in a molar proportion of 2.67:3.30:3.54:1.17:2.11:1.00. The monosaccharide contents of RIP-A1, RIP-B1 and RIP-B2 exhibited variations and none of them included GalA or GluA. Thus, it is possible that RIP-A1, RIP-B1 and RIP-B2 are neutral homopolysaccharides. Previous studies have reported that a neutral polysaccharide (RIWP) with a small M_w_ of 5.7 × 10^4^ Da, from *Isatis indigotica* Fort., was primarily made up of Glu, Gal and Ara [39]. Two fractions of pectin (WRIP-A-A and WRIP-A-B) and one neutral polysaccharide (WRIP-N) were obtained from the roots of *Isatis indigotica* Fort. Nevertheless, the compositions of WRIP-A-A and WRIP-A-B were elucidated, but WRIP-N remained unexplained [40]. Notably, a crude polysaccharide has been extracted from *Isatis indigotica* Fort., consisting of Gal, Glu, Fuc, Xyl, Ara, Man, GalA and GluA [45]. A relevant previous study suggests that RIP-A1, RIP-B1 and RIP-B2 are novel neutral polysaccharides from the roots of *Isatis indigotica* Fort. In addition, RIP-A1 exhibits the smallest M_w_ and has the simplest monosaccharide composition compared to RIP-B1 and RIP-B2.

### 2.3. Methylation Analysis

The glycosidic bonds and proportional molar ratios of RIP-A1, RIP-B1 and RIP-B2 were elucidated by methylation analysis (Table 1). The primary glycosidic linkages of RIP-A1 and RIP-B1 were identified as the 1,3,5-Araf and 1,6-Galp residues. This suggests that their glycan chains are linear and the branching point is at the O-3 position of the 1,3,5-Araf residue. In addition, small quantities of T-Arap and 1,5-Araf were found in RIP-A1, suggesting that T-Arap and 1,5-Araf are linked to arabinan side chains. T-Xylp and T-Arap were observed in RIP-B1, possibly as termini of arabinan side chains. However, RIP-B2 is mainly composed of 1,4-Glcp and 1,6-Galp residues, indicating a linear glycan chain. Moreover, small amounts of T-Xylp, T-Arap and 1,3,5-Araf were reflected in RIP-B2. These findings provide additional evidence that the structural characterization of RIP-A1, RIP-B1 and RIP-B2 shows variation during methylation analysis. Overall, the methylation results were consistent with the monosaccharide analyses. RIP-A1, RIP-B1 and RIP-B2 have similar glycosidic bond species. However, the specific arrangement of glycosidic bonds in RIP-A1 may be associated with the anti-inflammatory properties of neutral polysaccharides [46,47].

### 2.4. FT-IR Spectra

**Figure 3:**
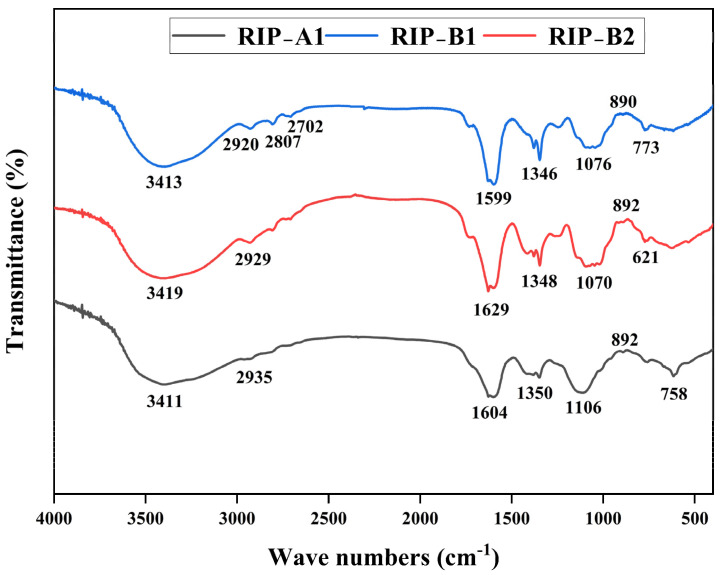
The FT-IR spectra of RIP-A1, RIP-B1 and RIP-B2.

As shown in Figure 3, RIP-A1, RIP-B1 and RIP-B2 each possess a prominent and wide absorption peak that results from the -OH stretching vibration in the polysaccharide structure (3000~3500 cm^−1^) [48]. The resonance of the C-H bond in the methyl group within the sugar ring (2800~3000 cm^−1^) produces the signal shown in the spectrum [49]. The absorption peaks are located at 1000~1200 cm^−1^, and they were found to originate from the stretching vibration of the C-O-H pendant group, the C-O-C glycosidic band vibration of the pyranose ring and the stretching vibration of the asymmetric ring, respectively [50]. The presence of these absorption peaks indicates that polysaccharides are present. The results suggest that the absorption peaks of RIP-A1, RIP-B1 and RIP-B2 exhibit high similarity. The carboxyl group of these polysaccharides does not exhibit C=O stretching, so they should be neutral polysaccharides [46]. In general, the results obtained from the FT-IR spectra are in agreement with the monosaccharide analysis.

### 2.5. NMR Analysis of RIP-A1

**Figure 4:**
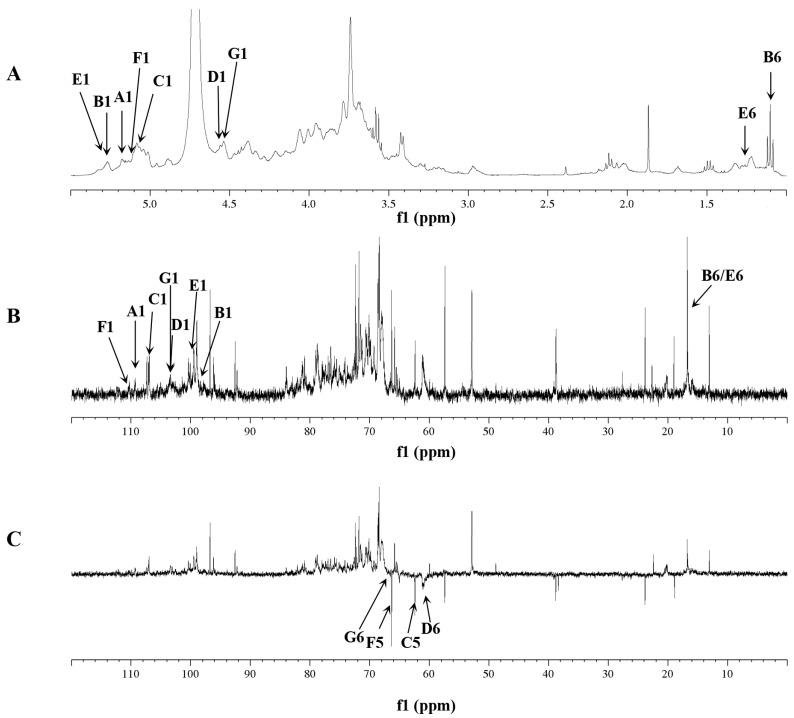
1D-NMR of RIP-A1. (**A**) ^1^H NMR spectra (400 MHz). (**B**) ^13^C NMR spectra (100 MHz). (**C**) DEPT-135 spectra (100 MHz).

RIP-A1 was subjected to further structural elucidation by ^1^H, ^13^C and DEPT-135 in conjunction with 2D NMR spectroscopy. The hydrogen and carbon signals are shown in Table 2. Figure 4A displays the ^1^H spectrum, which reveals the presence of anomeric protons at *δ* 5.16, 5.26, 5.07, 4.56, 5.01, 5.27 and 4.55. These protons indicate the existence of seven potential glycosidic residues (A–G) in RIP-A1. Combining the results of methylation analysis and other relevant data, T-Ara*f* (A), 1,2-Rha*p* (B), 1,5-Ara*f* (C), T-Gal*p* (D), 1,2,4-Rha*p* (E), 1,3,5-Ara*f* (F) and 1,6-Gal*p* (G) were tentatively confirmed [46,51,52]. Anomeric signals with *δ* > 5.0 ppm were generally recognized as α-configurations, whereas those with *δ* < 5.0 ppm were recognized as β-configurations [53]. The ^1^H spectrum revealed that A (*δ* 5.16), B (*δ* 5.26), C (*δ* 5.07), E-F (*δ* 5.01) and F (*δ* 5.27) were in *α*-configurations, but D (*δ* 4.56) and G (*δ* 4.55) were in *β*-configurations based on the anomeric signals.

**Figure 5:**
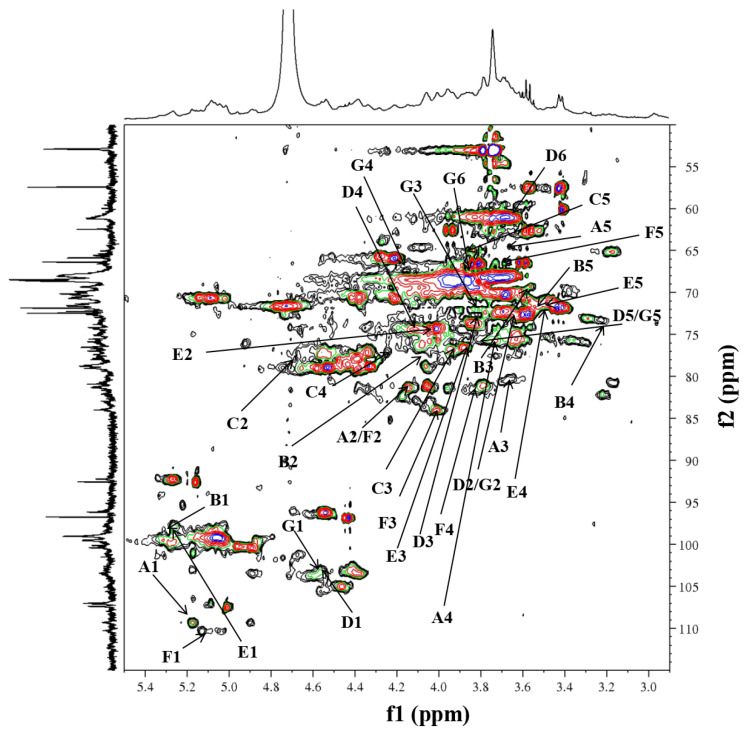
HSQC spectra of RIP-A1.

The anomeric signals (*δ*_H/C_) at 5.16/109.4, 5.26/98.4, 5.07/106.9, 4.56/103.4, 5.27/99.7, 5.13/110.5 and 4.55/103.5 corresponded to T-*α*-Ara*f*-(1→(A), →2)-*α*-Rha*p*-(1→(B), →5)-*α*-Ara*f*-(1→(C), T-*β*-Gal*p*-(1→(D), →2,4)-*α*-Rha*p*-(1→(E), →3,5)-*α*-Ara*f*-(1→(F) and →6)-*β*-Gal*p*-(1→(G) through the ^1^H, ^13^C (Figure 4A,B) and HSQC (Figure 5) spectra. In addition, the DEPT-135 spectrum (Figure 4C) was used for the assignment of some specific carbon signals of residues A, C, D, F and G. The inverted signals at *δ*_C_ 64.4, 65.1 and 66.5 corresponded to the C-5 of residues A, C and F. Then, the inverted signals at *δ*_C_ 61.0 and 66.4 were found to correlate with the C6 of residues D and G, respectively.

**Figure 6:**
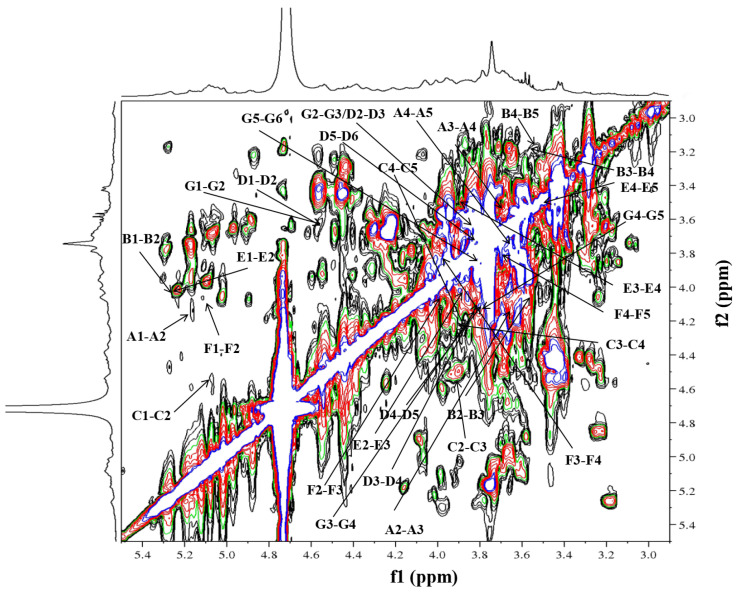
^1^H-^1^H COSY spectra of RIP-A1.

**Figure 7:**
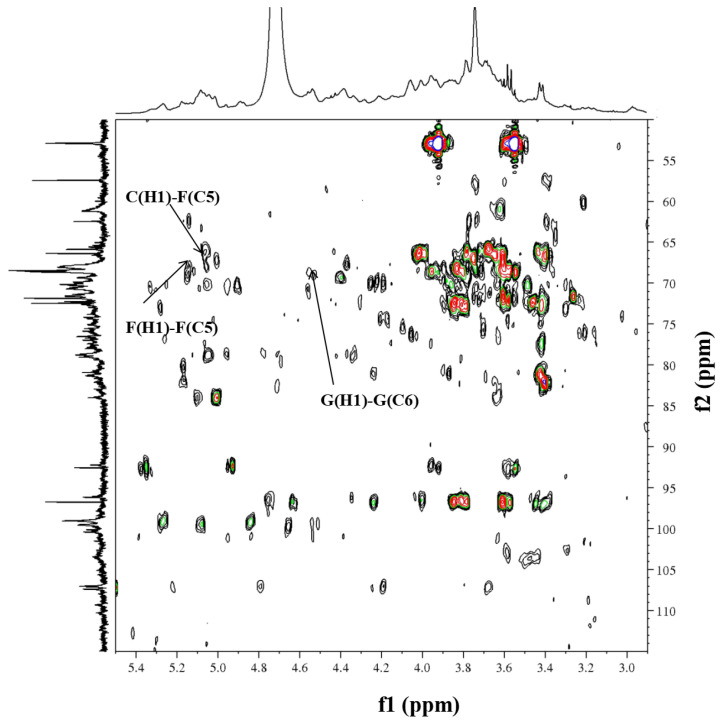
HMBC spectra of RIP-A1.

**Figure 8:**
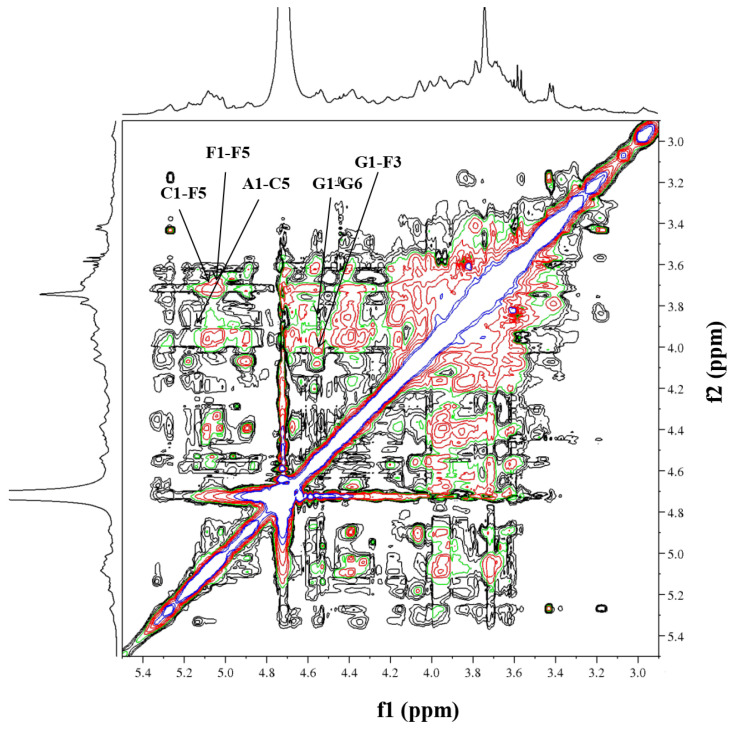
NOESY spectra of RIP-A1.

Initially, residue A exhibited an anomeric proton signal at *δ*_H_ 5.16. Further analysis of the ^1^H-^1^H COSY data (Figure 6) showed that the positions of its H2–H5 signals were at *δ*_H_ 4.15, 3.65, 3.75 and 3.66. Afterwards, the cross-peaks of C2–C6 were identified at *δ*_H_ 81.3, 80.4, 75.8 and 64.4 in the HSQC spectrum. The residue C displayed an anomeric proton signal at *δ*_H_ 5.07, and the chemical shift positions of H2–H5 at *δ*_H_ 4.53, 3.92, 4.24 and 3.86 were assigned by cross-peaks in the ^1^H-^1^H COSY spectrum. The ^13^C NMR chemical shifts of C2–C5 at *δ*_C_ 78.1, 76.7, 78.0 and 65.1 were assigned in the HSQC spectrum. The residue F, which had an anomeric proton signal at *δ*_H_ 5.13, showed cross-peaks between H2–H5 at *δ*_H_ 4.05, 3.99, 3.83 and 3.70 in the ^1^H-^1^H COSY spectrum. Next, the information on the main chain could be obtained from the HMBC (*δ*_H/C_) (Figure 7) and NOESY (*δ*_H/H_) (Figure 8) spectra. The correlated peaks A (H1)/C (C5) at *δ*_H/C_ 5.16/65.1, C (H1)/F (C5) at *δ*_H/C_ 5.07/66.5 and F (H1)/F (C5) at *δ*_H/C_ 5.13/66.5 indicate that a linear main chain is present as T-*α*-Ara*f*-(1→5)-*α*-Ara*f*-(1→5)-*α*-Ara*f*-(1→ and the branching point is arabinosyl at C-3, which concurs with the methylation analysis. Prior studies revealed comparable structural components in arabinan from *Ephedra sinica*, *Ligusticum chuanxiong* Hort. and *Rehmannia glutinosa* Libosch. However, differences were observed at the sites of the branching points [54,55,56].

Regarding residue D, the anomeric proton (*δ*_H_ 4.56) may be attributed to the H-1 of Gal*p*, which is related to the terminal position. According to the ^1^H-^1^H COSY spectrum, *δ*_H_ 3.63, 3.81, 4.11, 3.80, 3.67 and 3.78 were the chemical shift positions for H2 to H6. The ^13^C chemical shift locations were attributable to C-2 (*δ*_C_ 72.7), C-3 (*δ*_C_ 73.0), C-4 (*δ*_C_ 74.3), C-5 (*δ*_C_ 75.7) and C-6 (*δ*_C_ 61.0) in the HSQC spectrum. Moreover, the same data analysis method was used for the ^1^H and ^13^C of residue G, wherein the ^1^H and ^13^C NMR chemical shifts were *δ*_H-C_ 4.55/103.5, 3.63/72.9, 3.81/72.0, 4.16/66.7, 3.80/75.7 and 3.85, 3.78/68.4, respectively. Additionally, the chain of galactosan could be readily deduced by HMBC (*δ*_H/C_) and NOESY (*δ*_H/H_) correlations, e.g., D(H1)/G(C6) at *δ*_H/C_ 4.56/68.4, G(H1)/G(C6) at *δ*_H/C_ 4.55/68.4, D(H1)/G(H6) at *δ*_H/H_ 4.56/3.78 and D(H1)/G(H6) at *δ*_H/H_ 4.55/3.85. Thus, the inter-residual sequences of galactan were conjectured to be the occurrence of T-*β*-Gal*p*-(1→6)-*β*-Gal*p*-(1→6)-*β*-Gal*p*-(1→. 

It is noteworthy that some cross-peaks were investigated between G (H1) and F (H3) at *δ*_H/H_ 4.55/3.99, in the NOESY spectrum, suggesting that the galactan chains might be directly linked to the C-3 position of residue F. Previous research on traditional Chinese medicine has identified comparable structural fragments of neutral polysaccharides [57]. However, the presence of cross-peaks between residues B and E could not be verified due to their limited and unclear appearances in the HMBC and NOESY spectra. This outcome conforms with the methylation analysis. According to the aforementioned data, the primary composition of RIP-A1 consists of chains made up of arabinan (1,3,5-*α*-Ara*f*) and galactan (1,6-*β*-Gal*p*), with branching occurring at the C-3 position of the arabinosyl unit. 

Previous studies have revealed comparable structures in arabinogalactans obtained from plant polysaccharides, including HH1-1 (*Carthamus tinctorius* L.), LAG-W (*Larix kaempferi*) and LBGP70-OL (*Lycium barbarum*). For instance, the backbone of HH1-1 was found to be composed primarily of 1,6-linked Gal*p* branches at C-3. These branches predominantly contain 1,5-linked and 1,3,5-linked terminal arabinose and terminal galactose, as shown by the methylation and NMR analyses [58]. The arabinogalactan LAG-W was shown to possess a homogenous structure with a backbone composed of repetitive →3)-*β*-Gal*p*-(1→ residues. Additionally, it contains minor quantities of T-*α*-Ara*f*-(1→3)-*α*-Ara*f*-(1→6)-*β*-Gal*p*-(1→ residues [59]. The structural features of arabinogalactan from *Lycium barbarum* revealed that the backbone consists solely of →6)-*β*-Gal*p*-(1→ residues, which are replaced at the C3 position. The side chains, on the other hand, contain *α*-Ara*f*-(1→3)-*β*-Ara*f*-(1→3)-*β*-Ara*f*-(1→. However, several pectin structures were previously identified in the roots of *Isatis indigotica* Fort. This study primarily elucidates the structural characterization of arabinogalactan. 

### 2.6. SEM and AFM Analysis

**Figure 9:**
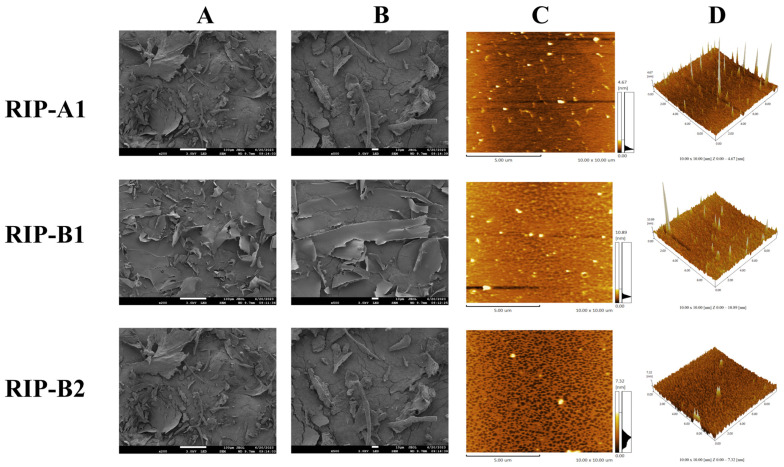
Surface morphology of RIP-A1, RIP-B1 and RIP-B2. (**A**) SEM image, 200× magnification. (**B**) SEM image, 500× magnification. (**C**) AFM image. (**D**) 3D topography.

RIP-A1, RIP-B1 and RIP-B2’s microstructures were investigated using a scanning electron microscope (SEM). The SEM images obtained with the three polysaccharides, of magnification (200- and 500-fold), are shown in Figure 9A,B. RIP-A1 exhibits a rough, wrinkled and uneven surface, with small voids and a honeycomb shape. RIP-B1 has a loosely arranged large lamellar structure, with slight folds on the surface and relatively flat edges. RIP-B2 has a smooth surface and flat edges, with a lamellar shape. Furthermore, the varying outcomes of RIP-A1, RIP-B1 and RIP-B2 may be linked to their differences in monosaccharide composition and strong intermolecular interactions [57,60,61]. 

Atomic force microscopy (AFM) has gained popularity recently as a tool for studying the chain structures of polysaccharide macromolecules. According to the data shown in Figure 9C,D, AFM analysis—conducted on a 2D floor plan—indicates that RIP-A1 displays a uniformly even polymer particle structure. RIP-B1 exhibits chain and irregular spherical conformations. RIP-B2 reveals a uniformly sized mesh conformation. The formation of polymer particles and sugar chains can be observed, which suggests that entangled states may exist in RIP-A1, RIP-B1 and RIP-B2 molecules [50,62]. Upon the examination of a 3D floor plan, it was seen that the surface of the polysaccharide is characterized by sharp peaks and a loose distribution. The molecular heights of RIP-A1 (2.08 nm), RIP-B1 (10.83 nm) and RIP-B2 (11.63 nm) were revealed by AFM measurements, while single-chain polysaccharides typically present heights of 0.1 to 1.0 nm. The intermolecular interactions, such as Van der Waals forces, acting between the individual polysaccharide chains might have led to their entanglement and formation of polymers. This entanglement may have caused the measured value to be larger than the actual value [63]. This might account for the discrepancy. The findings of RIP-A1, RIP-B1 and RIP-B2 exhibit variability, which may be related to differences in molecular weights. Previous research on the SEM and AFM of polysaccharides from the roots of *Isatis indigotica* Fort. is limited. In this study, the variations observed in the results of the three polysaccharides, when subjected to the same freeze-drying conditions, could potentially impact the isolation and purification process, as well as the polysaccharides themselves.

### 2.7. Anti-Inflammatory Activity Based on Raw 264.7 Cell Model

#### 2.7.1. Determination of Cytotoxicity

**Figure 10:**
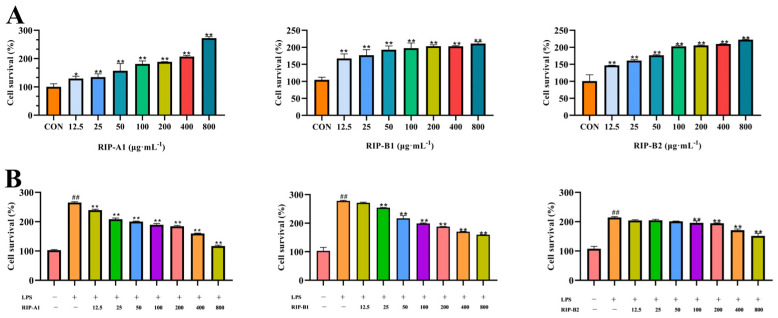
Effect of RIP-A1, RIP-B1 and RIP-B2 on cytotoxicity and activity in Raw 264.7 cells caused by LPS. (**A**) The effects of RIP-A1, RIP-B1 and RIP-B2 on the proliferation of RAW 264.7 cells ($\bar{x}$ ± s; *n* = 3) * *p* < 0.05, ** *p* < 0.01 vs. control. (**B**) The impacts of RIP-A1, RIP-B1 and RIP-B2 on activity in RAW 264.7 cells caused by LPS ($\bar{x}$ ± s; *n* = 3), ** *p* < 0.01 vs. the LPS-stimulated cells, ## *p* < 0.01 vs. control.

The influence of polysaccharides on the proliferation of RAW 264.7 cells was assessed by observing alterations in cell viability. According to Figure 10A, when cells were incubated for 24 h, RIP-A1, RIP-B1 and RIP-B2 did not induce cytotoxicity in RAW 264.7 cells. However, the cell viability was significantly increased (*p* < 0.05) in the concentration range of 12.5–800 μg·mL^−1^ compared to the control. This is because the presence of polysaccharides can induce the activation of protein kinase, which is important for cell proliferation, specifically in response to mitogens [64]. These data indicate that RIP-A1, RIP-B1 and RIP-B2 treatment does not cause cytotoxicity in RAW 264.7 cells.

#### 2.7.2. Screening of Anti-Inflammatory Activity of RIP-A1, RIP-B1 and RIP-B2

##### Effects of RIP-A1, RIP-B1 and RIP-B2 on RAW 264.7 Cell Activity Stimulated by LPS

LPS is an established immunomodulator that participates in several inflammatory reactions by causing the excessive production of specific inflammatory cytokines [65]. Initially, LPS was utilized to stimulate the cells for 24 h. After that, different concentrations of RIP-A1, RIP-B1 and RIP-B2 (50, 100, 200, 400 and 800 μg·mL^−1^) were introduced to the cells for another 24 h. The decreasing cell viability exhibited a positive correlation with the increasing concentrations of RIP-A1, RIP-B1 and RIP-B2 compared to the control. Figure 10B demonstrates that RIP-A1, RIP-B1 and RIP-B2 (at concentrations of 100, 200, 400 and 800 μg·mL^−1^) effectively inhibited the proliferation of LPS-activated RAW 264.7 cells. This inhibition was statistically significant (*p* < 0.01) and exhibited a dose-dependent relationship. These observations align with the overall trend observed in previously documented polysaccharides from the roots of *Isatis indigotica* Fort., which have demonstrated anti-inflammatory properties [39].

##### IL-4, IL- 1β, NO, TNF-α and IL-6 Determination

**Figure 11:**
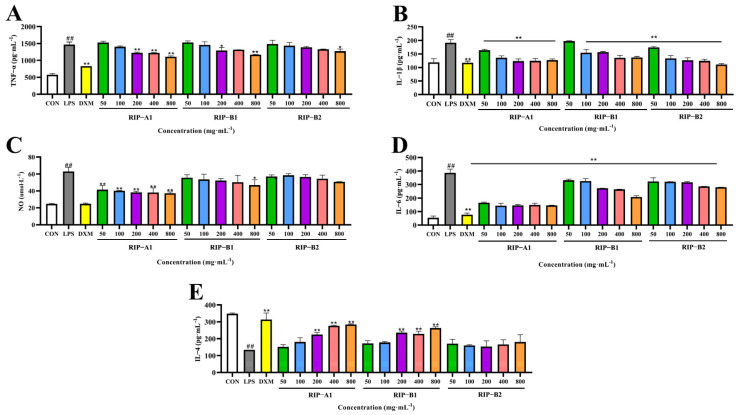
The effects of RIP-A1, RIP-B1 and RIP-B2 on the secretion of TNF-α, IL-1β, NO, IL-6 and IL-4 in RAW 264.7 cells. (**A**) TNF-α. (**B**) IL-1β. (**C**) NO. (**D**) IL-6. (**E**) IL-4. ($\bar{x}$ ± s; *n* = 3) * *p* < 0.05, ** *p* < 0.01 vs. the LPS-stimulated cells, ## *p* < 0.01 vs. control.

Immune and non-immune cells co-secrete intercellular signaling proteins known as cytokines, which regulate the body’s inflammatory response and immunity [46]. In contrast to the control group, the LPS group exhibited a significant increase in the IL-1β, IL-6, TNF-α and NO levels, accompanied by a significant decline in IL-4 levels (for all groups, *p* < 0.01), demonstrating that the LPS activation of the cells was successful in inducing an inflammatory response. As shown in Figure 11, the results reveal that RIP-A1, RIP-B1 and RIP-B2 suppressed the synthesis of IL-1β and IL-6 in a dose-dependent manner (range of 50 to 800 μg·mL^−1^). However, except for RIP-B2, all of them had a dose-dependent effect on the generation of IL-4. The NO content assay showed a significant decrease following treatment with RIP-A1 (*p* < 0.01) and RIP-B2 dosages of 400 and 800 ug·mL^−1^. Significant decreases in the TNF-α levels were observed at RIP-A1 doses of 200, 400 and 800 ug·mL^−1^ (*p* < 0.01). This research demonstrates that the molecular weights, mono-saccharide compositions, glycosidic linkages and conformations all impact the binding of receptors and the recognition of polysaccharide sites in inflammatory cells [66,67]. Polysaccharides with lower M_w_ are more likely to cross cell membranes and have biological effects [43]. Thus, RIP-A1, RIP-B1 and RIP-B2 may exhibit anti-inflammatory properties, varying based on their structural dissimilarities. The results demonstrate that RIP-A1 has the highest efficacy in terms of anti-inflammatory activities.

#### 2.7.3. Effect of RIP-A1 on RAW 264.7 Cells’ mRNA Expression of IL-1β, IL-6 and TNF-α Caused by LPS

**Figure 12:**
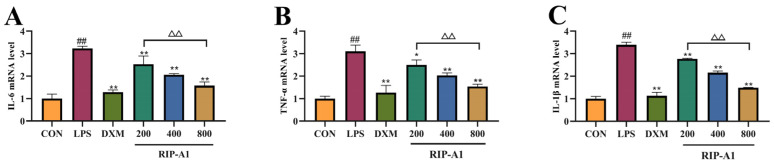
Expressions of IL-6, TNF-α and IL-1β mRNA in RAW 264.7 cells. (**A**) IL-6. (**B**) TNF-α (**C**) IL-1β. ($\bar{x}$ ± s; *n* = 3) * *p* < 0.05, ** *p* < 0.01 vs. the LPS-stimulated cells, ## *p* < 0.01 vs. control, ^ΔΔ^ *p* < 0.01 vs. comparison with 200 control group.

Cytokine synthesis is closely associated with mRNA expression and plays a vital role in intercellular communication in vivo. It is also essential for anti-inflammatory responses [46]. To investigate RIP-A1’s anti-inflammatory mechanism in RAW 264.7 cells, quantitative reverse-transcription PCR (qRT-PCR) was used to quantify the mRNA expression levels for IL-1β, IL-6 and TNF-α. As demonstrated in Figure 12, RIP-A1 exhibited dose-dependent inhibition of IL-1β, IL-6 and TNF-α mRNA expression. The LPS control group exhibited significantly elevated mRNA expression levels of IL-1β, IL-6 and TNF-α compared to the control group (*p* < 0.01), indicating the successful establishment of the inflammation model. In both DXM and RAW 264.7 cells, RIP-A1 reduced the mRNA levels of IL-6, IL-1β and TNF-α (*p* < 0.01) compared to the LPS control group. According to these results, LPS-induced RAW 264.7 cells’ expressions and secretions of IL-1β, IL-6 and TNF-α may be inhibited by RIP-A1.

#### 2.7.4. Effect of RIP-A1 on LPS-induced NF-κB Signaling Pathway in RAW 264.7 Cells

**Figure 13:**
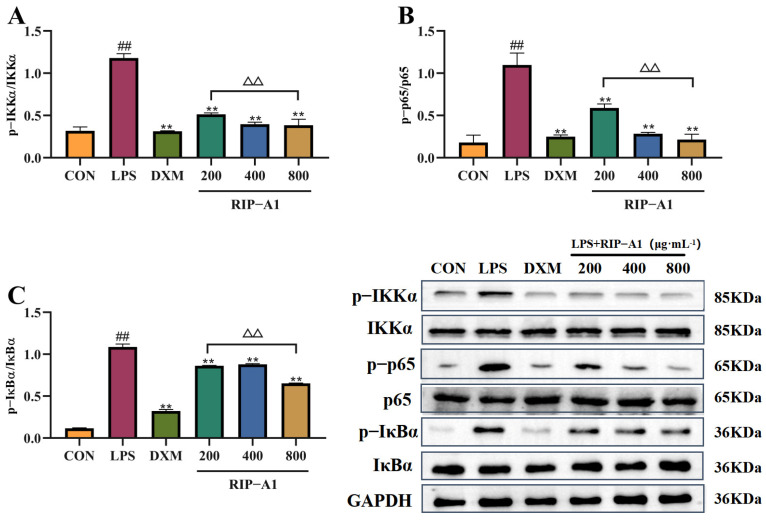
The p-IKKα, IKKα, p-p65, p65, p-IκBα and IκBα protein expression levels in RAW 264.7 cells. (**A**) p-IKKα and IKKα. (**B**) p-p65 and p65. (**C**) p-IκBα and IκBα. ($\bar{x}$ ± s; *n* = 3), ** *p* < 0.01 vs. the LPS-stimulated cells, ^##^ *p* < 0.01 vs. control, ^ΔΔ^ *p* < 0.01 vs. comparison with 200 control group.

**Figure 14:**
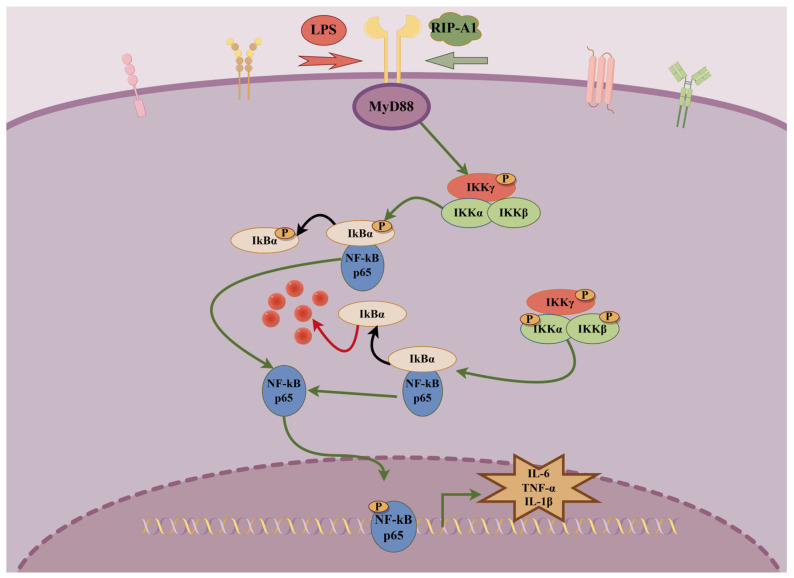
Diagram of the anti-inflammatory mechanism of RIP-A1.

Evidence has shown that LPS generated by Gram-negative (G^−^) bacteria can reach the bloodstream via penetrating compromised mucosal barriers, potentially activating the NF-κB signaling pathway [68]. Phosphorylating IKKα, p65 and IκBα may activate transcription factors through the activation of the NF-κB signaling pathway. Figure 13 illustrates the expression of the NF-kB signaling pathway, showing increased phosphorylation levels of IKKα, p65 and IκBα in response to LPS compared to the control group. The RIP-A1 treatment decreased the phosphorylation of IKKα, p65 and IκBα in a concentration-dependent manner. According to these results, the inhibition of phosphorylation by the receptor proteins mentioned earlier prevents the activation of the NF-κB signaling pathway, as well as the production of cytokines and NO. As shown in Figure 14, this may explain the anti-inflammatory properties of RIP-A1 on RAW 264.7 cells stimulated by LPS.

Previous studies have identified polysaccharides with comparable structures derived from several plants, many of which have anti-inflammatory properties. Examples include Lycium barbarum polysaccharide (LBP) and LAG-W (Larix kaempferi). For instance, the presence of large amounts of arabinose and galactose has a significant (*p* < 0.001) positive impact on the anti-inflammatory properties of the Lycium barbarum polysaccharide fraction (LBGP-I-3). This is achieved by increasing NO levels, enhancing phagocytosis and promoting acid phosphatase activity in RAW 264.7 cells. Additionally, the improved phagocytosis effect of LBGP-I-3 may be attributed to its ability to stimulate TLR4 receptors on the surfaces of macrophages. The addition of LAG-W significantly enhances the capacity of macrophages to engulf foreign particles while also triggering the release of NO and the cytokines TNF-α, IL-1β and IL-6. To summarize, the structural properties of arabinogalactan are associated with its significant anti-inflammatory effect [24,59,69].

## 3. Materials and Methods

### 3.1. Materials and Reagents

Radix isatidis plants were cultivated in Bajingzi Township, Datong District, Daqing City, Heilongjiang Province. D-Xylose (XYl), L-Rhamnose (Rha), L-Fucose (Fuc), D-Galactose (Gal), D-Ribose (Rib) and D-Arabinose (Ara) were acquired from Biotopped (Beijing, China). D-Mannose (Man) and D-Glucose (Glc) were acquired from Shanghai Yuanye Bio-Technology Co., Ltd. (Shanghai, China). Hexamethyl disilyamine (HMDS) and Chlorotrimethylsilane (TMCS) were purchased from Aladdin (Shanghai, China). ELISA kits were procured from Jiangsu Meimian Industrial Co., Ltd. (Jiangsu, China). Antibodies against p65, p-p65, IκBα, p-IκBα, IKKα and p-IKKα were obtained from ABclonal Technology Co., Ltd. (Wuhan, China). A BeyoRT™ III First Strand cDNA Synthesis Kit was procured from Beyotim (Shanghai, China). Unless otherwise noted, all chemicals and reagents were analytical grade. Distilled water was used in all experiments.

### 3.2. Exaction and Isolation of Polysaccharides 

The Radix isatidis were ground and defatted with 70% ethanol. Following that, deionized water was used to extract the residues. The water-based extracts were collected, filtrated, concentrated, precipitated, centrifugated and lyophilized. After being deproteinized using the Sevag-enzymatic process, the crude polysaccharides were given the designation RIP (*n* = 3) [70,71]. According to previous methods, the RIP (300 g) was dissolved in 5 L of dH_2_O and isolated by 20 KDa and 150 KDa ultrafiltration membranes (BONA-GM-20, BONA GROUP, Jinan, China) in sequence, and was designated as RIP-A–C. The DEAE-52 cellulose column (5 cm × 60 cm) was used to purify RIP-A1 in the amount of 1 mL·min^−1^ using a gradient elution NaCl solution (0–0.5 M) [47]. RIP-B was dissolved in dH_2_O and purified using a Sephadex G-100 column (2.6 cm × 100 cm), eluting with 0.1 M NaCl in the amount of 0.6 mL·min^−1^ [72]. Then these main fractions were concentrated and lyophilized to get purified polysaccharides (RIP-A1, RIP-B1 and RIP-B2). The content of sugar was measured using the phenol-sulfuric acid method [73].

### 3.3. Structural Analysis of Polysaccharides

#### 3.3.1. Molecular Weight Determination

The M_w_ determination was performed using a modified version of a previously reported protocol. The M_w_ of RIP was determined using a HPGPC system fitted with a column of Ultrahydrogel^TM^ Linear (7.8 mm × 300 mm, Waters, Milford, MA, USA) on an HPLC-evaporative light scattering detector (ELSD, Agilent 1260II HPLC, c.01.08.210, Agilent Technologies, Santa Clara, CA, USA). Distilled water was used as the mobile phase, with an injection volume of 10 μL and a flow rate of 0.8 mL·min^−1^. Standard molecular markers, T-series dextran (M_w_ = 270, 80, 50, 20 and 15 kDa), were employed (*n* = 3).

#### 3.3.2. Monosaccharide Composition Analysis

The monosaccharide compositions of polysaccharides were analyzed using methods based on previously reported work [44]. The mixed monosaccharide standards were added with NH_4_OH and kept at room temperature for 15 min. The procedure was completed by adding NaBH_4_, followed by securing the reaction vessel with a screw cover and letting the residue in the vial stand for 6 h at 25 °C. After that, the residue was dried by evaporating the NaBH_4_ and neutralized with acetic acid. The 5 mg of polysaccharides was dissolved in 2 M TFA and allowed to hydrolyze for 4 h at 110 °C in glass vials with caps. Following this, the hydrolysates were washed with methanol and repeatedly evaporated until completely dry to eliminate any remaining TFA. The hydrolyzed production and mixed monosaccharide standards were subjected to the same processes. The final solution was gently stirred and placed in a water bath at 70 °C for 30 min. Before GC–MS analysis, the solution was filtered via a 0.22 μm membrane. The following temperature conditions were applied to the TMSA derivatization process: 0 min at 180 °C, 4 °C·min^−1^ at 180–190 °C, 1 °C·min^−1^ at 190–200 °C, 2 °C·min^−1^ at 200–230 °C and 230–300 °C, followed by 5 min of holding.

#### 3.3.3. Infrared Spectra (IR) Analysis

To prepare the samples for infrared spectrum analysis, the freeze-dried RIP-A1, RIP-B1 and RIP-B2 were crushed with dry KBr powder and pressed into pellets. The FT-IR spectrum of polysaccharides was acquired by a Fourier-transform infrared spectrograph (VERTEX-70, FTR 5.4.9, Bruker, Rheinstetten, Germany) at frequencies between 500 and 4000 cm^−1^ to obtain structural data.

#### 3.3.4. Methylation Analysis

According to the earlier approach, the freeze-dried RIP-A1, RIP-B1 and RIP-B2 (2 mg) were completely methylated and were hydrolyzed, reduced, acetylated and analyzed using a GC–MS system (Agilent 7890A-5975C, 10.0, Agilent Technologies, Santa Clara, CA, USA) that was fitted with a DB-5 silica capillary column (60 m × 0.25 mm × 0.25 μm) [74]. The following temperature conditions were applied: 80 °C for 1 min, 80–200 °C at 5 °C·min^−1^, 200–220 °C at 2 °C·min^−1^ and 220–270 °C at 10 °C·min^−1^ and a 5 min hold time.

#### 3.3.5. NMR Analysis

The RIP-A1 was dissolved in D_2_O for NMR analysis. 1D/2D-NMR spectra were registered on an NMR spectrometer (Bruker AM-400, 11.1, Bruker, Fällanden, Switzerland).

#### 3.3.6. SEM and AFM Analysis

The surface characterization of RIP-A1, RIP-B1 and RIP-B2 was performed by SEM (JSM-7800F, B17.0.07, JEOL Ltd., Tokyo, Japan). Dried RIP-A1, RIP-B1 and RIP-B2 were secured with electrical tapes and coated using a vacuum spraying device. An acceleration voltage was applied while the constructed sample was operated under a vacuum. 

Following the dissolution of RIP-A1, RIP-B1 and RIP-B2 in distilled water with a concentration of 5 µg·mL^−1^, the resulting mixture was passed through a cellulose membrane (0.22 μm). After applying the sample solution over a freshly cleaved mica, drying was performed at room temperature. The AFM analysis was conducted using SPM-9700 apparatus (SPM-9700, 2.61, Shimadzu, Kyoto, Japan). Working conditions were set to a scanning range of 1.000 × 1.000 μm with a scanning frequency of 1.00 Hz and tapping mode was selected.

### 3.4. Anti-Inflammatory Activity on RAW 264.7 Cells

#### 3.4.1. Determination of Cytotoxicity

Briefly, 10^5^ RAW 264.7 cells were cultivated into each well of a 96-well plate and incubated [38]. The cells were allowed to adhere for 24 h. After that, the cells were treated with different concentrations of RIP-A1, RIP-B1 and RIP-B2 (formulated as 12.5, 25, 50, 100, 200, 400 and 800 μg·mL^−1^) for 24 h. After adding 10% CCK-8 solution, the cells were incubated for 2 h. Using a microplate reader, the cell viability was determined at 450 nm (SpectraMax ABS Plus, 7.1.0, Molecular Devices, San Jose, CA, USA).

#### 3.4.2. Screening of Anti-Inflammatory Activity of RIP-A1, RIP-B1 and RIP-B2

##### Effects of RIP-A1, RIP-B1 and RIP-B2 on LPS-Induced Activity of RAW 264.7 Cells

RAW 264.7 cells were cultured and treated with LPS (1 μg·mL^−1^) for 24 h to induce inflammation [39,75]. The cells were cultured with various doses of RIP-A1, RIP-B1 and RIP-B2 (25, 50, 100, 200, 400 and 800 μg·mL^−1^). The LPS group consisted of cells only treated with LPS, while the control group consisted of RAW 264.7 cells with complete medium only. A microplate reader (SpectraMax ABS Plus, 7.1.0, Molecular Devices, San Jose, CA, USA) was then used to measure the absorbance at 450 nm.

##### Effects of RIP-A1, RIP-B1 and RIP-B2 on LPS-induced IL-4, IL-1β, NO, TNF-α and IL-6 Production in RAW 264.7 Cells

The experiment was set up with a blank control group, an LPS control group, a positive control group (10 μg·mL^−1^ of dexamethasone, DXM) and the polysaccharide group (final concentrations of 50, 100, 200, 400 and 800 μg·mL^−1^). The 48-well plates containing RAW 264.7 cells (10^5^ cells/well) were incubated for 24 h at 37 °C in a humidified incubator with 5% CO_2_. Cells were treated with LPS (except for the normal group), then the supernatant was collected and IL-1β, IL-6, TNF-α, NO and IL-4 levels were assessed using an ELISA kit according to the provided instructions [61].

#### 3.4.3. qRT-PCR Analysis

The extraction of total RNA from RAW 264.7 cells, including differentiated cells with and without RIP-A1, was performed using TRIzol reagent following the directions provided by the manufacturer. A cDNA reverse transcription kit was used to get cDNA from 1 μg RNA from each sample. The mRNA expression was measured by qPCR under the following reaction conditions: 40 cycles of PCR amplification, pre-denaturation at 95 °C for 2 min, denaturation at 95 °C for 15 s and annealing and extension at 60 °C for 30 s. By normalizing the Ct data to GAPDH expression, the comparative CT (2^−ΔΔCt^) approach was used to assess the relative gene expression. Table 3 contains a list of the primer set sequences employed for amplification [76]. 

#### 3.4.4. Western Blotting Analysis

The Western blotting technique was employed, following the methods used in a previously reported experiment with minor modifications [38]. Radioimmunoprecipitation assay (RIPA) buffer was utilized to extract the whole protein content from RAW 264.7 cells. Subsequently, a bicinchoninic acid (BCA) kit was employed to measure the protein concentrations in these lysates. After separating proteins using sodium dodecyl-sulfate polyacrylamide gel electrophoresis (SDS-PAGE), the proteins were transferred onto polyvinylidene fluoride (PVDF) membranes and incubated overnight with antibodies specific to p65, p-p65, IκBα, p-IκBα, IKKα and p-IKKα at 4 °C. The membranes were then washed with tris-buffered saline with Tween-20 (TBST). An ODYSSEY CLx Gel Imaging System (Tanon-4200, 2.6.0.0, Tanon, Shanghai, China) was used to image the resultant blots.

### 3.5. Data Analysis 

Multiple comparison tests and one-way analysis of variance (ANOVA) were used to evaluate the data. The data were presented as the means ± standard errors of the means ($\bar{x}$ ± s). Differences were considered significant when *p* < 0.05 and highly significant when *p* < 0.01. GraphPad Prism 8.0 (GraphPad Software, Boston, MA, USA) and Origin 2022 were used for graphic data processing.

## 4. Conclusions

To summarize, this study extracted and isolated three novel polysaccharides (RIP-A1, RIP-B1 and RIP-B2) from the roots of *Isatis indigotica* Fort. The primary structures of RIP-A1, RIP-B1 and RIP-B2 were identified by chemical and spectral methods, indicating that they are neutral polysaccharides with different M_w_ and monosaccharide compositions. In addition, RIP-A1 (M_w_ = 1.8 × 10^4^ Da) was determined to be a smaller M_w_ arabinogalactan with glycosidic linkages of T-*α*-Ara*f*, 1,2-*α*-Rha*p*, 1,5-*α*-Ara*f*, T-*β*-Gal*p*, 1,2,4-*α*-Rha*p*, 1,3,5-*α*-Ara*f* and 1,6-*β*-Gal*p*. However, RIP-B1 (M_w_ = 7.4 × 10^4^ Da) and RIP-B2 (M_w_ = 9.3 × 10^4^ Da) are more complex neutral polysaccharides consisting of Xyl or Glc. The structural features and differences between RIP-A1, RIP-B1 and RIP-B2 could affect their anti-inflammation activity. The anti-inflammation activities of RIP-A1, RIP-B1 and RIP-B2 in RAW 264.7 cells were evaluated. In contrast to RIP-B1 and RIP-B2, RIP-A1 exhibited the best anti-inflammatory activity in vitro. The results indicate that RIP-A1 has a considerable inhibitory effect on the production of pro-inflammatory cytokines within specific concentration ranges. It also reduces the mRNA levels of IL-1β, IL-6 and TNF-α and downregulates the expressions of p-IKKα, p-p65 and p-IκBα. Therefore, it may be speculated that RIP-A1 demonstrates an anti-inflammatory impact by stimulating the NF-κB signaling pathway. In future investigations, the focus should be on investigating the connection between RIP-A1 and cellular receptors. This work presents a rigorous theoretical basis for the structure–activity relationship of RIP-A1 and reveals its potential as a natural anti-inflammatory compound with therapeutic properties.

## Figures and Tables

**Table 1:** The glycosidic linkage types and molar percentages of RIP-A1, RIP-B1 and RIP-B2 based on the methylation and GC–MS analyses.

Type of Glycosidic Bond	PMAA	Mass Fragments (*m*/*z*)	Mol (%)		
			RIP-A1	RIP-B1	RIP-B2
T-Ara*p*	1,5-di-O-Ac-2,3,4-tri-O-Me Ara	59, 71, 87, 102, 118, 129, 145	0.33	3.2	0.30
1,5-Ara*f*	1,4,5-tri-O-Ac-2,3-di-O-Me Ara	59, 71, 87, 102, 118, 129, 142, 189	1.02	0.36	0.01
1,3,5-Ara*f*	1,3,4,5-tet-O-Ac-2-O-Me Ara	74, 85, 99, 118, 127, 141, 159, 173	11.41	8.9	0.80
1,2-Rha*p*	1,2,5-tri-O-Ac-3,4-di-O-Me Man	57, 85, 115, 131, 190, 281, 304	0.12	-	-
1,2,4-Rha*p*	1,2,4,5-tet-O-Ac-3-O-Me Man	59, 74, 88, 101, 130, 143, 190, 203	0.45	-	-
T-Gal*p*	1,5-di-O-Ac-2,3,4,6-tet-O-Me Gal	59, 74, 88, 101, 130, 143, 190, 203	0.09	-	-
1,6-Gal*p*	1,5,6-tri-O-Ac-2,3,4-tri-O-Me Gal	59, 71, 88, 99, 117, 129, 157	10.23	9.6	4.60
T-Xyl*p*	1,5-di-O-Ac-2,3,4-tri-O-Me Xyl	59, 73, 87, 101, 115, 129	-	2.8	0.77
1,4-Fuc*p*	1,4,5-tri-O-Ac-2,3-di-O-Me Gal	59, 72, 87, 101, 118, 129, 143	-	0.01	0.01
1,4-Glc*p*	1,4,5-tri-O-Ac-2,3,6-tri-O-Me Glc	59, 71, 99, 117, 129, 142	-	-	7.51

Ac: acetyl; Me: methyl.

**Table 2:** ^1^H/^13^C chemical shifts of RIP-A1 from HSQC data.

Sugar Residues	Chemical Shifts, *δ* (ppm)					
	H-1/C-1	H-2/C-2	H-3/C-3	H-4/C-4	H-5/C-5	H-6/C-6
(A) T-*α*-Ara*f*-(1→	5.16/109.4	4.15/81.3	3.65/80.4	3.75/75.8	3.66/64.4	
(B) →2)-*α*-Rha*p*-(1→	5.26/98.4	4.06/76.7	3.56/69.7	3.18/75.8	3.53/75.7	1.10/16.7
(C) →5)-*α*-Ara*f*-(1→	5.07/106.9	4.53/78.1	3.92/76.7	4.24/78.0	3.86/65.1	
(D) T-*β*-Galp-(1→	4.56/103.4	3.63/72.7	3.81/73.0	4.11/74.3	3.80/75.7	3.67, 3.78/61.0
(E) →2,4)-*α*-Rha*p*-(1→	5.27/99.7	4.04/74.1	3.88/76.6	3.50/72.7	3.52/71.8	1.25/16.7
(F) →3,5)-*α*-Ara*f*-(1→	5.13/110.5	4.05/81.1	3.99/84.1	3.83/81.1	3.70/66.5	
(G) →6)-*β*-Gal*p*-(1→	4.55/103.5	3.63/72.9	3.81/72.0	4.16/66.7	3.8075.7	3.85, 3.78/68.4

**Table 3:** Real-time quantitative PCR primer sequences.

Gene	Primary Sequence
IL-6	Forward:5′-CCAAGAGGTGAGTGCTTCCC-3′
	Reverse:5′-CTGTTGTTCAGACTCTCTCCCT-3′
IL-1β	Forward:5′-GACGTGGAACTGGCAGAAGAG-3
	Reverse:5′-TTGGTGGTTTGTGAGTGTGAG-3
TNF-α	Forward:5′-GCAACTGTTCCTGAACTCAACT-3
	Reverse:5′-ATCTTTTGGGGTCCGTCAACT-3
β-action	Forward:5′-GTGCTATGTTGCTCTAGACTTCG-3
	Reverse:5′-ATGCCACAGGATTCCA-TACC-3

## Data Availability

Data are contained within the article.

## Supplementary Materials

The following supporting information can be downloaded at: https://www.mdpi.com/article/10.3390/molecules29112683/s1, Figure S1. Elution profiles. (A) Elution curves of RIP-A1-5 components. (B) Elution curves of RIP-B1-2 components. Figure S2. Molecular weight of RIP-A1, RIP-B1 and RIP-B2. (A) HPLC chromatogram of RIP-A1. (B) HPLC chromatogram of RIP-B1. (C) HPLC chromatogram of RIP-B2.

## Abbreviations

RIP, Radix Isatidis polysaccharides; HPGPC, high-performance gel permeation chromatography; HPLC, high-performance liquid chromatography; GC–MS, gas chromatography–mass spectrometry; TMSA, trimethylsilyl-alditol; Ara, arabinose; Rha, rhamnose; Gal, galactose; Xyl, xylose; Fuc, fucose; Rib, ribose; Glc, glucose; GalA, galacturonic acid; Man, mannose; GluA, glucuronic acid; SEM, scanning electron microscope; AFM, atomic force microscopy; qRT-PCR, quantitative reverse-transcription PCR; HMDS, hexamethyl disilyamine; ELSD, evaporative light scattering detector; DXMS, dexamethasone; RIPA buffer, radioimmunoprecipitation assay buffer; BCA, bicinchoninic acid; SDS-PAGE, sodium dodecyl-sulfate polyacrylamide gel electrophoresis; PVDF, polyvinylidene fluoride; TBST, tris-buffered saline with Tween-20; LPS, lipopolysaccharide; TLR4, toll-like receptor 4; NF-κB, nuclear factor κappaβ; M_w_, molecular weight; FT-IR, Fourier-transform infrared; TMCS, chlorotrimethylsilane.
