# Correction: Shen et al. The Isolation, Structural Characterization and Anti-Inflammatory Potentials of Neutral Polysaccharides from the Roots of *Isatis indigotica* Fort. *Molecules* 2024, *29*, 2683

**DOI:** 10.3390/molecules29214987

**Published:** 2024-10-22

**Authors:** Yu Shen, Shihao Wu, Mingming Song, Huiming Zhang, Hong Zhao, Lili Wu, Hongbo Zhao, Hongbin Qiu, Yu Zhang

**Affiliations:** 1 Heilongjiang Provincial Key Laboratory of New Drug Development and Pharmacotoxicological Evaluation, College of Pharmacy, Jiamusi University, Jiamusi 154007, China; shenyu@jmsu.edu.cn (Y.S.); 228153048@stu.jmsu.edu.cn (S.W.); 15765339879@163.com (M.S.); banana_5016@163.com (H.Z.); zhaohong1981@jmsu.edu.cn (H.Z.); h42003@163.com (L.W.); 2 College of Rehabilitation Medicine, Jiamusi University, Jiamusi 154007, China; zhaohongbo@jmsu.edu.cn

Errors in Figure

In the original publication [1], there were mistakes in Figure 9. The A and B images of RIP-A1 and RIP-B2 in Figure 9 were duplicated (SEM result images). The C and D images of RIP-A1, RIP-B1, and RIP-B2 in Figure 9 were in the incorrect order (AFM result images). The corrected Figure 9 appears below. The authors state that the scientific conclusions are unaffected. This correction was approved by the Academic Editor. The original publication has also been updated.

## Figures and Tables

**Figure 9:**
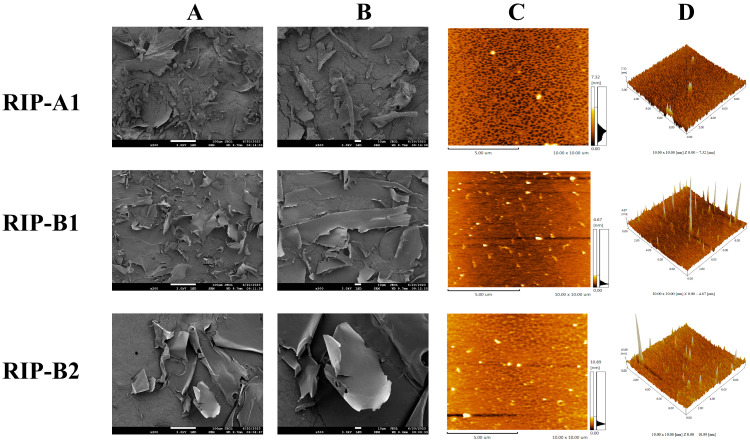
Surface morphology of RIP-A1, RIP-B1 and RIP-B2. (**A**) SEM image, 200× magnification. (**B**) SEM image, 500× magnification. (**C**) AFM image. (**D**) 3D topography.
