# Supplementary material for: The Isolation, Structural Characterization and Anti-Inflammatory Potentials of Neutral Polysaccharides from the Roots of Isatis indigotica Fort
Source: Molecules. 2024 Jun 5;29(11):2683. doi: 10.3390/molecules29112683 (PMC11173581; doi:10.3390/molecules29112683)
Supplement: Supplementary file 1 [file molecules-29-02683-s001.zip › molecules-3010588-supplementary.pdf]

The Isolation, Structural Characterization and Anti-Inflammatory Potentials of Neutral Polysaccharides from the Roots of *Isatis indigotica* Fort.

Yu Shen [1], Shihao Wu [1], Mingming Song [1], Huiming Zhang [1], Hong Zhao [1], Lili Wu [1], Hongbo Zhao [2], Hongbin Qiu [1,*] and Yu Zhang [1,*]

[1] Heilongjiang Provincial Key Laboratory of New Drug Development and Pharmacotoxicological Evaluation, College of Pharmacy, Jiamusi University, Jiamusi 154007, China; shenyu@jmsu.edu.cn (Y.S.); 228153048@stu.jmsu.edu.cn (S.W.); 15765339879@163.com (M.S.); banana_5016@163.com (H.Z.); zhaohong1981@jmsu.edu.cn (H.Z.); h42003@163.com (L.W.)

[2] College of Rehabilitation Medicine, Jiamusi University, Jiamusi 154007, China; zhaohongbo@jmsu.edu.cn

* Correspondence: qhbin63@163.com (H.Q.); zhangyu@jmsu.edu.cn (Y.Z.)

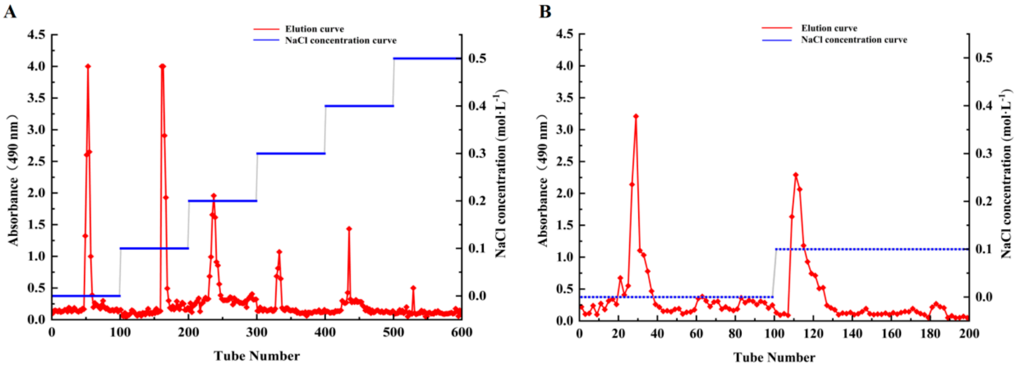

Figure S1. Elution profiles. (A) Elution curves of RIP-A1-5 components. (B) Elution curves of RIP-B1-2 components.

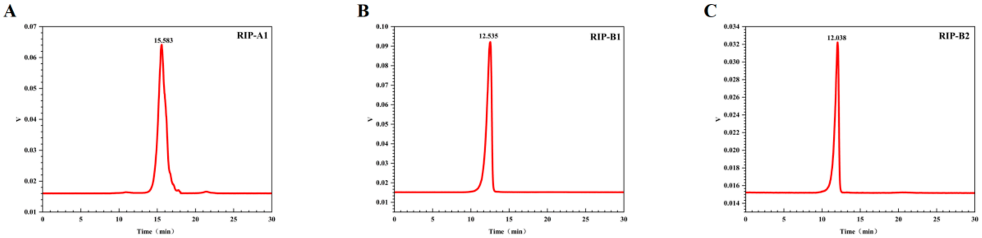

Figure S2. Molecular weight of RIP-A1, RIP-B1 and RIP-B2. (A) HPLC chromatogram of RIP-A1. (B) HPLC chromatogram of RIP-B1. (C) HPLC chromatogram of RIP-B2.
